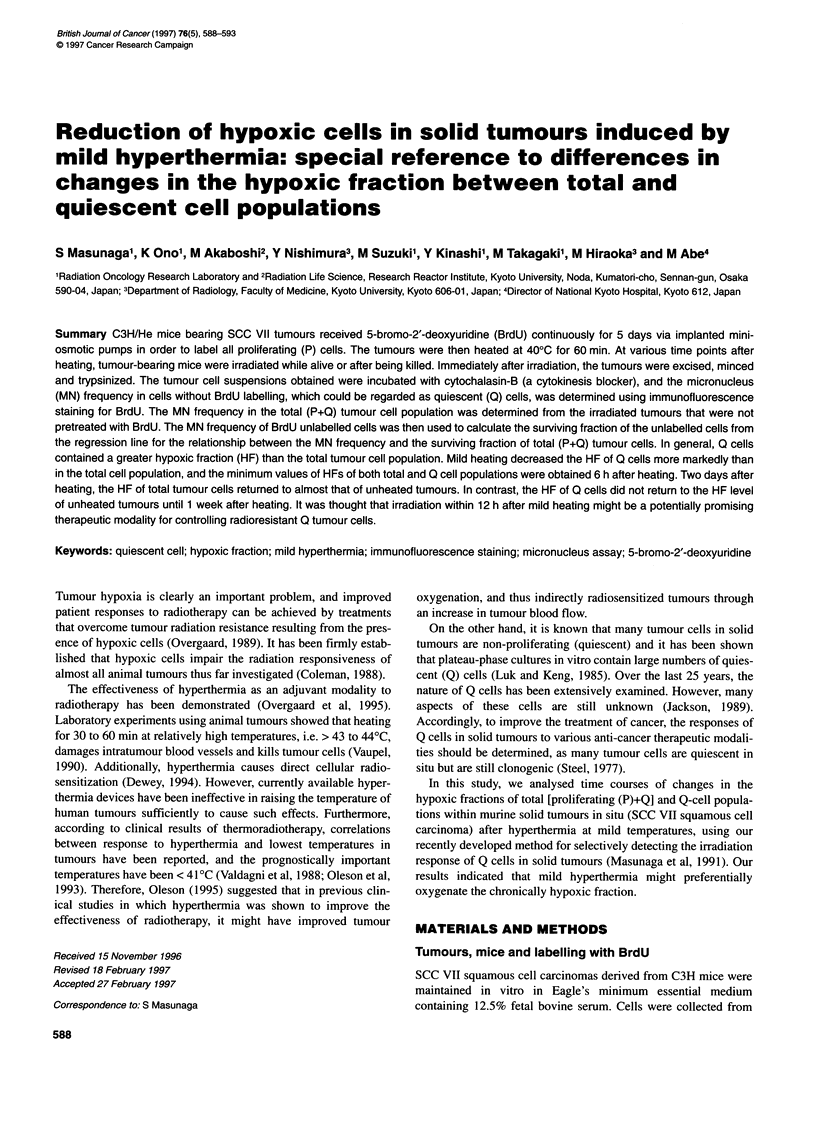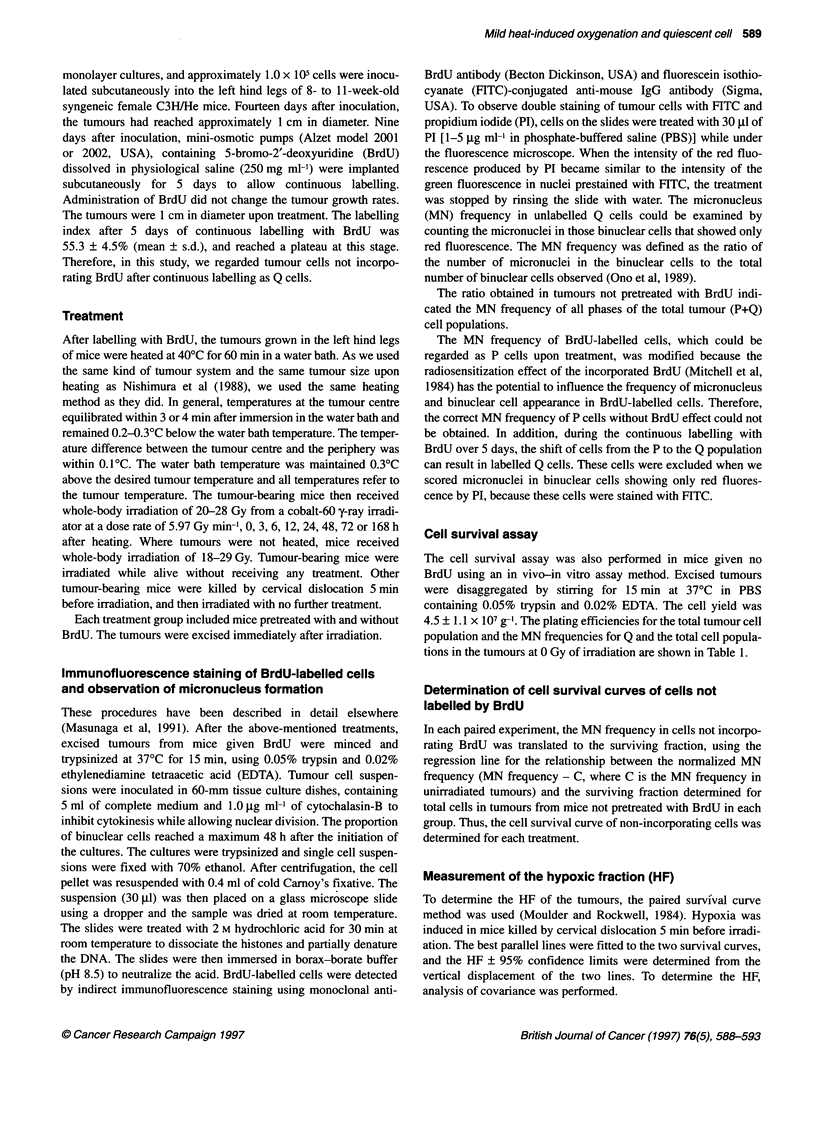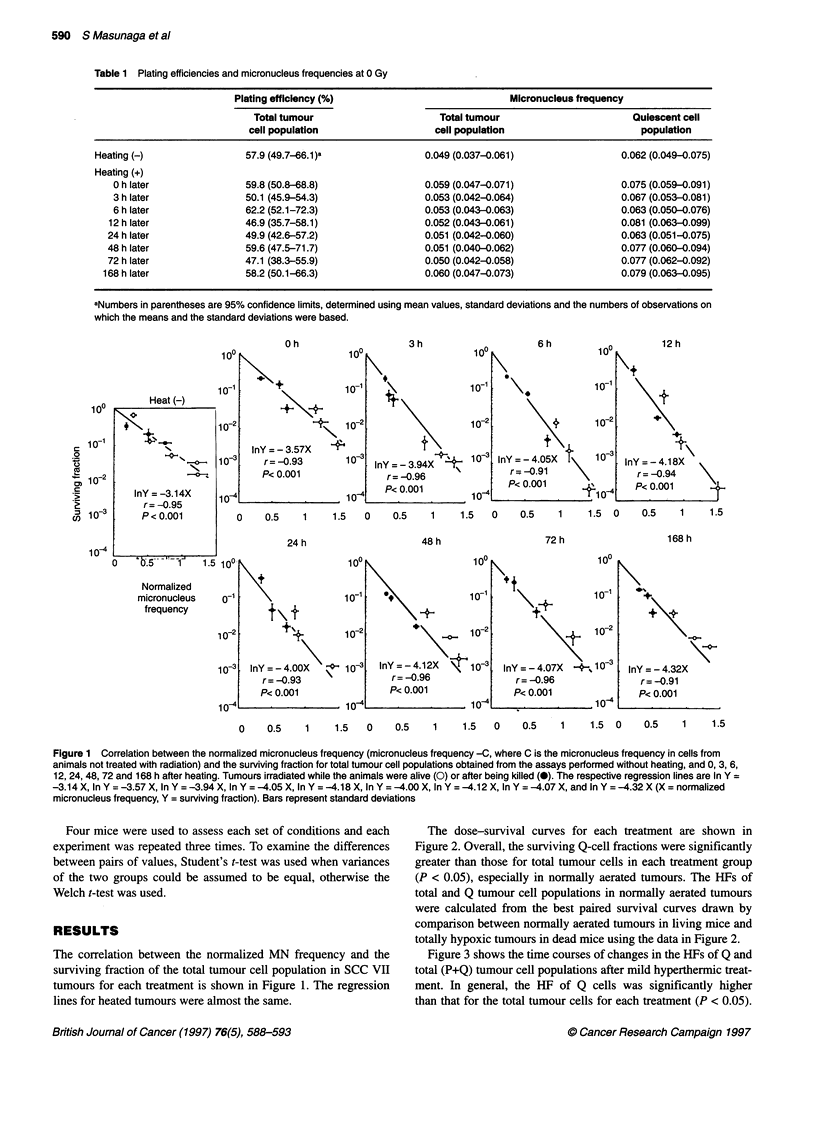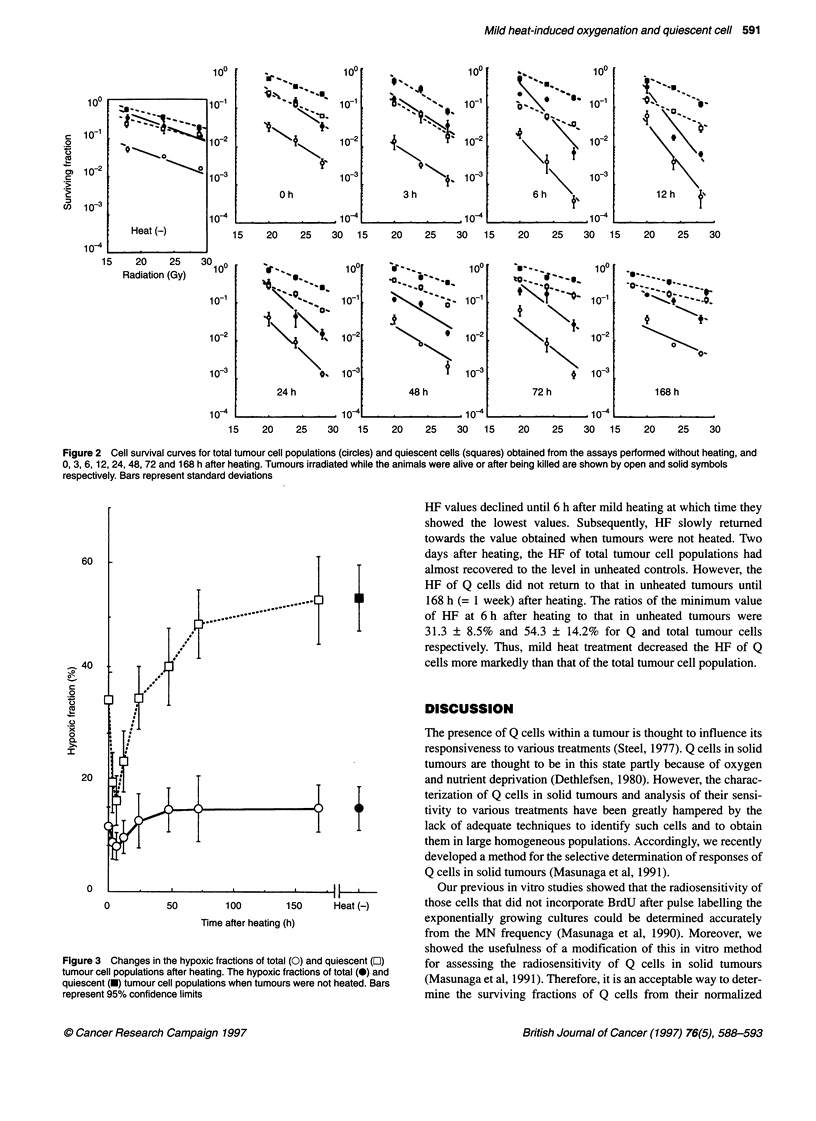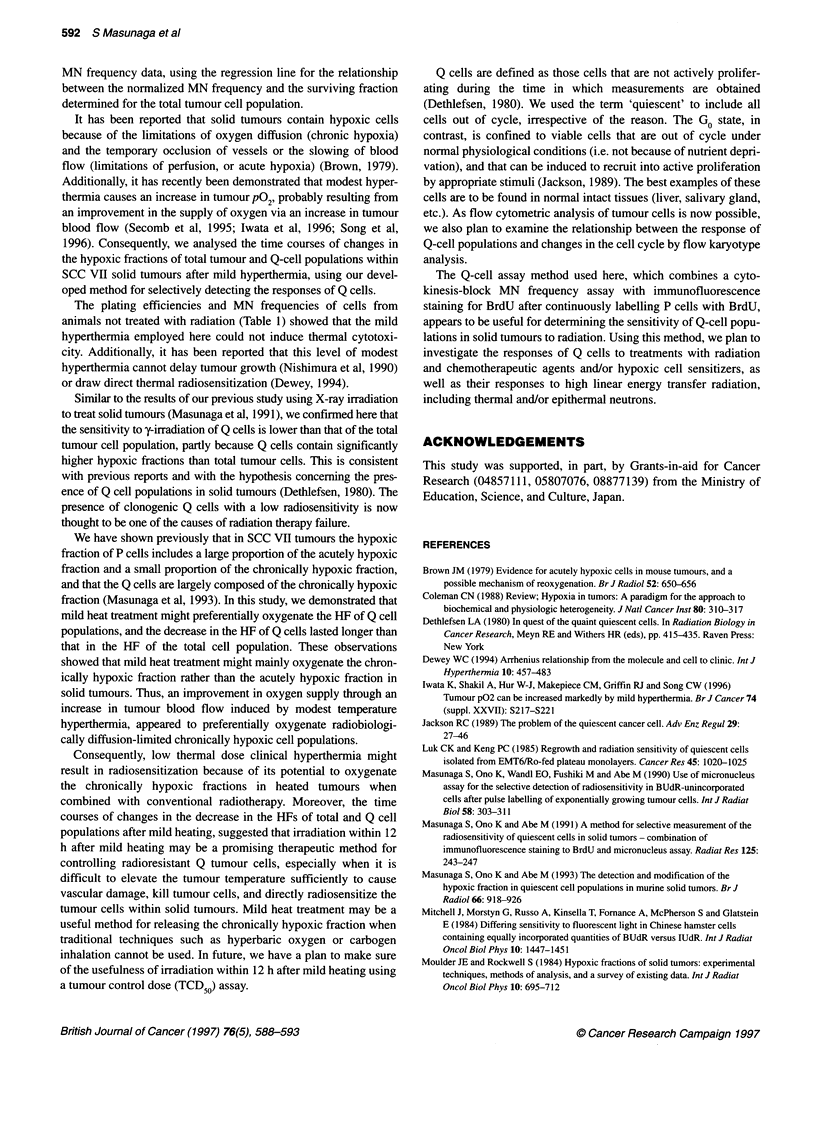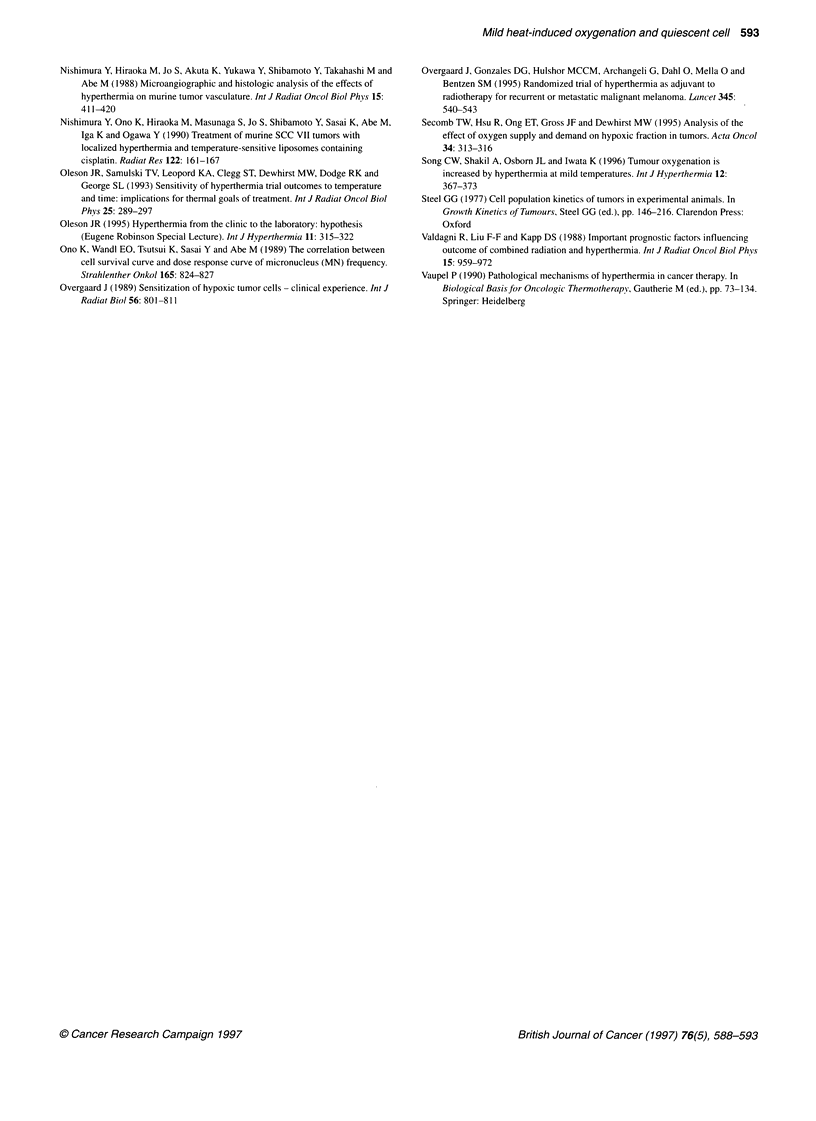# Reduction of hypoxic cells in solid tumours induced by mild hyperthermia: special reference to differences in changes in the hypoxic fraction between total and quiescent cell populations.

**DOI:** 10.1038/bjc.1997.430

**Published:** 1997

**Authors:** S. Masunaga, K. Ono, M. Akaboshi, Y. Nishimura, M. Suzuki, Y. Kinashi, M. Takagaki, M. Hiraoka, M. Abe

**Affiliations:** Radiation Oncology Research Laboratory, Kyoto University, Osaka, Japan.

## Abstract

C3H/He mice bearing SCC VII tumours received 5-bromo-2'-deoxyuridine (BrdU) continuously for 5 days via implanted mini-osmotic pumps in order to label all proliferating (P) cells. The tumours were then heated at 40 degrees C for 60 min. At various time points after heating, tumour-bearing mice were irradiated while alive or after being killed. Immediately after irradiation, the tumours were excised, minced and trypsinized. The tumour cell suspensions obtained were incubated with cytochalasin-B (a cytokinesis blocker), and the micronucleus (MN) frequency in cells without BrdU labelling, which could be regarded as quiescent (Q) cells, was determined using immunofluorescence staining for BrdU. The MN frequency in the total (P+Q) tumour cell population was determined from the irradiated tumours that were not pretreated with BrdU. The MN frequency of BrdU unlabelled cells was then used to calculate the surviving fraction of the unlabelled cells from the regression line for the relationship between the MN frequency and the surviving fraction of total (P+Q) tumour cells. In general, Q cells contained a greater hypoxic fraction (HF) than the total tumour cell population. Mild heating decreased the HF of Q cells more markedly than in the total cell population, and the minimum values of HFs of both total and Q cell populations were obtained 6 h after heating. Two days after heating, the HF of total tumour cells returned to almost that of unheated tumours. In contrast, the HF of Q cells did not return to the HF level of unheated tumours until 1 week after heating. It was thought that irradiation within 12 h after mild heating might be a potentially promising therapeutic modality for controlling radioresistant Q tumour cells.


British Journal of Cancer (1997) 76(5), 588–593
© 1997 Cancer Research Campaign

### Reduction of hypoxic cells in solid tumours induced by mild hyperthermia: special reference to differences in changes in the hypoxic fraction between total and quiescent cell populations

**S Masunaga[1], K Ono[1], M Akaboshi[2], Y Nishimura[3], M Suzuki[1], Y Kinashi[1], M Takagaki[1], M Hiraoka[3] and M Abe[4]**

[1]Radiation Oncology Research Laboratory and [2]Radiation Life Science, Research Reactor Institute, Kyoto University, Noda, Kumatori-cho, Sennan-gun, Osaka 590-04, Japan; [3]Department of Radiology, Faculty of Medicine, Kyoto University, Kyoto 606-01, Japan; [4]Director of National Kyoto Hospital, Kyoto 612, Japan

**Summary** C3H/He mice bearing SCC VII tumours received 5-bromo-2′-deoxyuridine (BrdU) continuously for 5 days via implanted mini-osmotic pumps in order to label all proliferating (P) cells. The tumours were then heated at 40°C for 60 min. At various time points after heating, tumour-bearing mice were irradiated while alive or after being killed. Immediately after irradiation, the tumours were excised, minced and trypsinized. The tumour cell suspensions obtained were incubated with cytochalasin-B (a cytokinesis blocker), and the micronucleus (MN) frequency in cells without BrdU labelling, which could be regarded as quiescent (Q) cells, was determined using immunofluorescence staining for BrdU. The MN frequency in the total (P+Q) tumour cell population was determined from the irradiated tumours that were not pretreated with BrdU. The MN frequency of BrdU unlabelled cells was then used to calculate the surviving fraction of the unlabelled cells from the regression line for the relationship between the MN frequency and the surviving fraction of total (P+Q) tumour cells. In general, Q cells contained a greater hypoxic fraction (HF) than the total tumour cell population. Mild heating decreased the HF of Q cells more markedly than in the total cell population, and the minimum values of HFs of both total and Q cell populations were obtained 6 h after heating. Two days after heating, the HF of total tumour cells returned to almost that of unheated tumours. In contrast, the HF of Q cells did not return to the HF level of unheated tumours until 1 week after heating. It was thought that irradiation within 12 h after mild heating might be a potentially promising therapeutic modality for controlling radioresistant Q tumour cells.

**Keywords:** quiescent cell; hypoxic fraction; mild hyperthermia; immunofluorescence staining; micronucleus assay; 5-bromo-2′-deoxyuridine

Tumour hypoxia is clearly an important problem, and improved patient responses to radiotherapy can be achieved by treatments that overcome tumour radiation resistance resulting from the presence of hypoxic cells (Overgaard, 1989). It has been firmly established that hypoxic cells impair the radiation responsiveness of almost all animal tumours thus far investigated (Coleman, 1988).

The effectiveness of hyperthermia as an adjuvant modality to radiotherapy has been demonstrated (Overgaard et al, 1995). Laboratory experiments using animal tumours showed that heating for 30 to 60 min at relatively high temperatures, i.e. > 43 to 44°C, damages intratumour blood vessels and kills tumour cells (Vaupel, 1990). Additionally, hyperthermia causes direct cellular radiosensitization (Dewey, 1994). However, currently available hyperthermia devices have been ineffective in raising the temperature of human tumours sufficiently to cause such effects. Furthermore, according to clinical results of thermoradiotherapy, correlations between response to hyperthermia and lowest temperatures in tumours have been reported, and the prognostically important temperatures have been < 41°C (Valdagni et al, 1988; Oleson et al, 1993). Therefore, Oleson (1995) suggested that in previous clinical studies in which hyperthermia was shown to improve the effectiveness of radiotherapy, it might have improved tumour oxygenation, and thus indirectly radiosensitized tumours through an increase in tumour blood flow.

On the other hand, it is known that many tumour cells in solid tumours are non-proliferating (quiescent) and it has been shown that plateau-phase cultures in vitro contain large numbers of quiescent (Q) cells (Luk and Keng, 1985). Over the last 25 years, the nature of Q cells has been extensively examined. However, many aspects of these cells are still unknown (Jackson, 1989). Accordingly, to improve the treatment of cancer, the responses of Q cells in solid tumours to various anti-cancer therapeutic modalities should be determined, as many tumour cells are quiescent in situ but are still clonogenic (Steel, 1977).

In this study, we analysed time courses of changes in the hypoxic fractions of total [proliferating (P)+Q] and Q-cell populations within murine solid tumours in situ (SCC VII squamous cell carcinoma) after hyperthermia at mild temperatures, using our recently developed method for selectively detecting the irradiation response of Q cells in solid tumours (Masunaga et al, 1991). Our results indicated that mild hyperthermia might preferentially oxygenate the chronically hypoxic fraction.

### MATERIALS AND METHODS

### Tumours, mice and labelling with BrdU

SCC VII squamous cell carcinomas derived from C3H mice were maintained in vitro in Eagle's minimum essential medium containing 12.5% fetal bovine serum. Cells were collected from

*Received 15 November 1996*
*Revised 18 February 1997*
*Accepted 27 February 1997*

*Correspondence to:* S Masunaga monolayer cultures, and approximately $1.0 \times 10^5$ cells were inoculated subcutaneously into the left hind legs of 8- to 11-week-old syngeneic female C3H/He mice. Fourteen days after inoculation, the tumours had reached approximately 1 cm in diameter. Nine days after inoculation, mini-osmotic pumps (Alzet model 2001 or 2002, USA), containing 5-bromo-2′-deoxyuridine (BrdU) dissolved in physiological saline (250 mg $ml^{-1}$) were implanted subcutaneously for 5 days to allow continuous labelling. Administration of BrdU did not change the tumour growth rates. The tumours were 1 cm in diameter upon treatment. The labelling index after 5 days of continuous labelling with BrdU was 55.3 ± 4.5% (mean ± s.d.), and reached a plateau at this stage. Therefore, in this study, we regarded tumour cells not incorporating BrdU after continuous labelling as Q cells.

### Treatment

After labelling with BrdU, the tumours grown in the left hind legs of mice were heated at 40°C for 60 min in a water bath. As we used the same kind of tumour system and the same tumour size upon heating as Nishimura et al (1988), we used the same heating method as they did. In general, temperatures at the tumour centre equilibrated within 3 or 4 min after immersion in the water bath and remained 0.2–0.3°C below the water bath temperature. The temperature difference between the tumour centre and the periphery was within 0.1°C. The water bath temperature was maintained 0.3°C above the desired tumour temperature and all temperatures refer to the tumour temperature. The tumour-bearing mice then received whole-body irradiation of 20–28 Gy from a cobalt-60 γ-ray irradiator at a dose rate of 5.97 Gy $min^{-1}$, 0, 3, 6, 12, 24, 48, 72 or 168 h after heating. Where tumours were not heated, mice received whole-body irradiation of 18–29 Gy. Tumour-bearing mice were irradiated while alive without receiving any treatment. Other tumour-bearing mice were killed by cervical dislocation 5 min before irradiation, and then irradiated with no further treatment.

Each treatment group included mice pretreated with and without BrdU. The tumours were excised immediately after irradiation.

### Immunofluorescence staining of BrdU-labelled cells and observation of micronucleus formation

These procedures have been described in detail elsewhere (Masunaga et al, 1991). After the above-mentioned treatments, excised tumours from mice given BrdU were minced and trypsinized at 37°C for 15 min, using 0.05% trypsin and 0.02% ethylenediamine tetraacetic acid (EDTA). Tumour cell suspensions were inoculated in 60-mm tissue culture dishes, containing 5 ml of complete medium and 1.0 μg $ml^{-1}$ of cytochalasin-B to inhibit cytokinesis while allowing nuclear division. The proportion of binuclear cells reached a maximum 48 h after the initiation of the cultures. The cultures were trypsinized and single cell suspensions were fixed with 70% ethanol. After centrifugation, the cell pellet was resuspended with 0.4 ml of cold Carnoy's fixative. The suspension (30 μl) was then placed on a glass microscope slide using a dropper and the sample was dried at room temperature. The slides were treated with 2 M hydrochloric acid for 30 min at room temperature to dissociate the histones and partially denature the DNA. The slides were then immersed in borax–borate buffer (pH 8.5) to neutralize the acid. BrdU-labelled cells were detected by indirect immunofluorescence staining using monoclonal anti-BrdU antibody (Becton Dickinson, USA) and fluorescein isothiocyanate (FITC)-conjugated anti-mouse IgG antibody (Sigma, USA). To observe double staining of tumour cells with FITC and propidium iodide (PI), cells on the slides were treated with 30 μl of PI [1–5 μg $ml^{-1}$ in phosphate-buffered saline (PBS)] while under the fluorescence microscope. When the intensity of the red fluorescence produced by PI became similar to the intensity of the green fluorescence in nuclei prestained with FITC, the treatment was stopped by rinsing the slide with water. The micronucleus (MN) frequency in unlabelled Q cells could be examined by counting the micronuclei in those binuclear cells that showed only red fluorescence. The MN frequency was defined as the ratio of the number of micronuclei in the binuclear cells to the total number of binuclear cells observed (Ono et al, 1989).

The ratio obtained in tumours not pretreated with BrdU indicated the MN frequency of all phases of the total tumour (P+Q) cell populations.

The MN frequency of BrdU-labelled cells, which could be regarded as P cells upon treatment, was modified because the radiosensitization effect of the incorporated BrdU (Mitchell et al, 1984) has the potential to influence the frequency of micronucleus and binuclear cell appearance in BrdU-labelled cells. Therefore, the correct MN frequency of P cells without BrdU effect could not be obtained. In addition, during the continuous labelling with BrdU over 5 days, the shift of cells from the P to the Q population can result in labelled Q cells. These cells were excluded when we scored micronuclei in binuclear cells showing only red fluorescence by PI, because these cells were stained with FITC.

### Cell survival assay

The cell survival assay was also performed in mice given no BrdU using an in vivo–in vitro assay method. Excised tumours were disaggregated by stirring for 15 min at 37°C in PBS containing 0.05% trypsin and 0.02% EDTA. The cell yield was $4.5 \pm 1.1 \times 10^7$ $g^{-1}$. The plating efficiencies for the total tumour cell population and the MN frequencies for Q and the total cell populations in the tumours at 0 Gy of irradiation are shown in Table 1.

### Determination of cell survival curves of cells not labelled by BrdU

In each paired experiment, the MN frequency in cells not incorporating BrdU was translated to the surviving fraction, using the regression line for the relationship between the normalized MN frequency (MN frequency – C, where C is the MN frequency in unirradiated tumours) and the surviving fraction determined for total cells in tumours from mice not pretreated with BrdU in each group. Thus, the cell survival curve of non-incorporating cells was determined for each treatment.

### Measurement of the hypoxic fraction (HF)

To determine the HF of the tumours, the paired survival curve method was used (Moulder and Rockwell, 1984). Hypoxia was induced in mice killed by cervical dislocation 5 min before irradiation. The best parallel lines were fitted to the two survival curves, and the HF ± 95% confidence limits were determined from the vertical displacement of the two lines. To determine the HF, analysis of covariance was performed.

© Cancer Research Campaign 1997

**Table 1** Plating efficiencies and micronucleus frequencies at 0 Gy

| | Plating efficiency (%) | Micronucleus frequency | |
|---|---|---|---|
| | Total tumour cell population | Total tumour cell population | Quiescent cell population |
| Heating (–) | 57.9 (49.7–66.1)[a] | 0.049 (0.037–0.061) | 0.062 (0.049–0.075) |
| Heating (+) | | | |
| 0 h later | 59.8 (50.8–68.8) | 0.059 (0.047–0.071) | 0.075 (0.059–0.091) |
| 3 h later | 50.1 (45.9–54.3) | 0.053 (0.042–0.064) | 0.067 (0.053–0.081) |
| 6 h later | 62.2 (52.1–72.3) | 0.053 (0.043–0.063) | 0.063 (0.050–0.076) |
| 12 h later | 46.9 (35.7–58.1) | 0.052 (0.043–0.061) | 0.081 (0.063–0.099) |
| 24 h later | 49.9 (42.6–57.2) | 0.051 (0.042–0.060) | 0.063 (0.051–0.075) |
| 48 h later | 59.6 (47.5–71.7) | 0.051 (0.040–0.062) | 0.077 (0.060–0.094) |
| 72 h later | 47.1 (38.3–55.9) | 0.050 (0.042–0.058) | 0.077 (0.062–0.092) |
| 168 h later | 58.2 (50.1–66.3) | 0.060 (0.047–0.073) | 0.079 (0.063–0.095) |

[a]Numbers in parentheses are 95% confidence limits, determined using mean values, standard deviations and the numbers of observations on which the means and the standard deviations were based.

**Figure 1** Correlation between the normalized micronucleus frequency (micronucleus frequency –C, where C is the micronucleus frequency in cells from animals not treated with radiation) and the surviving fraction for total tumour cell populations obtained from the assays performed without heating, and 0, 3, 6, 12, 24, 48, 72 and 168 h after heating. Tumours irradiated while the animals were alive (○) or after being killed (●). The respective regression lines are ln Y = –3.14 X, ln Y = –3.57 X, ln Y = –3.94 X, ln Y = –4.05 X, ln Y = –4.18 X, ln Y = –4.00 X, ln Y = –4.12 X, ln Y = –4.07 X, and ln Y = –4.32 X (X = normalized micronucleus frequency, Y = surviving fraction). Bars represent standard deviations

Four mice were used to assess each set of conditions and each experiment was repeated three times. To examine the differences between pairs of values, Student's *t*-test was used when variances of the two groups could be assumed to be equal, otherwise the Welch *t*-test was used.

### RESULTS

The correlation between the normalized MN frequency and the surviving fraction of the total tumour cell population in SCC VII tumours for each treatment is shown in Figure 1. The regression lines for heated tumours were almost the same.

The dose–survival curves for each treatment are shown in Figure 2. Overall, the surviving Q-cell fractions were significantly greater than those for total tumour cells in each treatment group ($P < 0.05$), especially in normally aerated tumours. The HFs of total and Q tumour cell populations in normally aerated tumours were calculated from the best paired survival curves drawn by comparison between normally aerated tumours in living mice and totally hypoxic tumours in dead mice using the data in Figure 2.

Figure 3 shows the time courses of changes in the HFs of Q and total (P+Q) tumour cell populations after mild hyperthermic treatment. In general, the HF of Q cells was significantly higher than that for the total tumour cells for each treatment ($P < 0.05$).

 © Cancer Research Campaign 1997

**Figure 2** Cell survival curves for total tumour cell populations (circles) and quiescent cells (squares) obtained from the assays performed without heating, and 0, 3, 6, 12, 24, 48, 72 and 168 h after heating. Tumours irradiated while the animals were alive or after being killed are shown by open and solid symbols respectively. Bars represent standard deviations

**Figure 3** Changes in the hypoxic fractions of total (○) and quiescent (□) tumour cell populations after heating. The hypoxic fractions of total (●) and quiescent (■) tumour cell populations when tumours were not heated. Bars represent 95% confidence limits

HF values declined until 6 h after mild heating at which time they showed the lowest values. Subsequently, HF slowly returned towards the value obtained when tumours were not heated. Two days after heating, the HF of total tumour cell populations had almost recovered to the level in unheated controls. However, the HF of Q cells did not return to that in unheated tumours until 168 h (= 1 week) after heating. The ratios of the minimum value of HF at 6 h after heating to that in unheated tumours were 31.3 ± 8.5% and 54.3 ± 14.2% for Q and total tumour cells respectively. Thus, mild heat treatment decreased the HF of Q cells more markedly than that of the total tumour cell population.

### DISCUSSION

The presence of Q cells within a tumour is thought to influence its responsiveness to various treatments (Steel, 1977). Q cells in solid tumours are thought to be in this state partly because of oxygen and nutrient deprivation (Dethlefsen, 1980). However, the characterization of Q cells in solid tumours and analysis of their sensitivity to various treatments have been greatly hampered by the lack of adequate techniques to identify such cells and to obtain them in large homogeneous populations. Accordingly, we recently developed a method for the selective determination of responses of Q cells in solid tumours (Masunaga et al, 1991).

Our previous in vitro studies showed that the radiosensitivity of those cells that did not incorporate BrdU after pulse labelling the exponentially growing cultures could be determined accurately from the MN frequency (Masunaga et al, 1990). Moreover, we showed the usefulness of a modification of this in vitro method for assessing the radiosensitivity of Q cells in solid tumours (Masunaga et al, 1991). Therefore, it is an acceptable way to determine the surviving fractions of Q cells from their normalized

© Cancer Research Campaign 1997

MN frequency data, using the regression line for the relationship between the normalized MN frequency and the surviving fraction determined for the total tumour cell population.

It has been reported that solid tumours contain hypoxic cells because of the limitations of oxygen diffusion (chronic hypoxia) and the temporary occlusion of vessels or the slowing of blood flow (limitations of perfusion, or acute hypoxia) (Brown, 1979). Additionally, it has recently been demonstrated that modest hyperthermia causes an increase in tumour $pO_2$, probably resulting from an improvement in the supply of oxygen via an increase in tumour blood flow (Secomb et al, 1995; Iwata et al, 1996; Song et al, 1996). Consequently, we analysed the time courses of changes in the hypoxic fractions of total tumour and Q-cell populations within SCC VII solid tumours after mild hyperthermia, using our developed method for selectively detecting the responses of Q cells.

The plating efficiencies and MN frequencies of cells from animals not treated with radiation (Table 1) showed that the mild hyperthermia employed here could not induce thermal cytotoxicity. Additionally, it has been reported that this level of modest hyperthermia cannot delay tumour growth (Nishimura et al, 1990) or draw direct thermal radiosensitization (Dewey, 1994).

Similar to the results of our previous study using X-ray irradiation to treat solid tumours (Masunaga et al, 1991), we confirmed here that the sensitivity to $\gamma$-irradiation of Q cells is lower than that of the total tumour cell population, partly because Q cells contain significantly higher hypoxic fractions than total tumour cells. This is consistent with previous reports and with the hypothesis concerning the presence of Q cell populations in solid tumours (Dethlefsen, 1980). The presence of clonogenic Q cells with a low radiosensitivity is now thought to be one of the causes of radiation therapy failure.

We have shown previously that in SCC VII tumours the hypoxic fraction of P cells includes a large proportion of the acutely hypoxic fraction and a small proportion of the chronically hypoxic fraction, and that the Q cells are largely composed of the chronically hypoxic fraction (Masunaga et al, 1993). In this study, we demonstrated that mild heat treatment might preferentially oxygenate the HF of Q cell populations, and the decrease in the HF of Q cells lasted longer than that in the HF of the total cell population. These observations showed that mild heat treatment might mainly oxygenate the chronically hypoxic fraction rather than the acutely hypoxic fraction in solid tumours. Thus, an improvement in oxygen supply through an increase in tumour blood flow induced by modest temperature hyperthermia, appeared to preferentially oxygenate radiobiologically diffusion-limited chronically hypoxic cell populations.

Consequently, low thermal dose clinical hyperthermia might result in radiosensitization because of its potential to oxygenate the chronically hypoxic fractions in heated tumours when combined with conventional radiotherapy. Moreover, the time courses of changes in the decrease in the HFs of total and Q cell populations after mild heating, suggested that irradiation within 12 h after mild heating may be a promising therapeutic method for controlling radioresistant Q tumour cells, especially when it is difficult to elevate the tumour temperature sufficiently to cause vascular damage, kill tumour cells, and directly radiosensitize the tumour cells within solid tumours. Mild heat treatment may be a useful method for releasing the chronically hypoxic fraction when traditional techniques such as hyperbaric oxygen or carbogen inhalation cannot be used. In future, we have a plan to make sure of the usefulness of irradiation within 12 h after mild heating using a tumour control dose ($TCD_{50}$) assay.

Q cells are defined as those cells that are not actively proliferating during the time in which measurements are obtained (Dethlefsen, 1980). We used the term 'quiescent' to include all cells out of cycle, irrespective of the reason. The $G_0$ state, in contrast, is confined to viable cells that are out of cycle under normal physiological conditions (i.e. not because of nutrient deprivation), and that can be induced to recruit into active proliferation by appropriate stimuli (Jackson, 1989). The best examples of these cells are to be found in normal intact tissues (liver, salivary gland, etc.). As flow cytometric analysis of tumour cells is now possible, we also plan to examine the relationship between the response of Q-cell populations and changes in the cell cycle by flow karyotype analysis.

The Q-cell assay method used here, which combines a cytokinesis-block MN frequency assay with immunofluorescence staining for BrdU after continuously labelling P cells with BrdU, appears to be useful for determining the sensitivity of Q-cell populations in solid tumours to radiation. Using this method, we plan to investigate the responses of Q cells to treatments with radiation and chemotherapeutic agents and/or hypoxic cell sensitizers, as well as their responses to high linear energy transfer radiation, including thermal and/or epithermal neutrons.

### ACKNOWLEDGEMENTS

This study was supported, in part, by Grants-in-aid for Cancer Research (04857111, 05807076, 08877139) from the Ministry of Education, Science, and Culture, Japan.

### REFERENCES

Brown JM (1979) Evidence for acutely hypoxic cells in mouse tumours, and a possible mechanism of reoxygenation. *Br J Radiol* **52**: 650–656

Coleman CN (1988) Review; Hypoxia in tumors: A paradigm for the approach to biochemical and physiologic heterogeneity. *J Natl Cancer Inst* **80**: 310–317

Dethlefsen LA (1980) In quest of the quaint quiescent cells. In *Radiation Biology in Cancer Research*, Meyn RE and Withers HR (eds), pp. 415–435. Raven Press: New York

Dewey WC (1994) Arrhenius relationship from the molecule and cell to clinic. *Int J Hyperthermia* **10**: 457–483

Iwata K, Shakil A, Hur W-J, Makepiece CM, Griffin RJ and Song CW (1996) Tumour pO2 can be increased markedly by mild hyperthermia. *Br J Cancer* **74** (suppl. XXVII): S217–S221

Jackson RC (1989) The problem of the quiescent cancer cell. *Adv Enz Regul* **29**: 27–46

Luk CK and Keng PC (1985) Regrowth and radiation sensitivity of quiescent cells isolated from EMT6/Ro-fed plateau monolayers. *Cancer Res* **45**: 1020–1025

Masunaga S, Ono K, Wandl EO, Fushiki M and Abe M (1990) Use of micronucleus assay for the selective detection of radiosensitivity in BUdR-unincorporated cells after pulse labelling of exponentially growing tumour cells. *Int J Radiat Biol* **58**: 303–311

Masunaga S, Ono K and Abe M (1991) A method for selective measurement of the radiosensitivity of quiescent cells in solid tumors – combination of immunofluorescence staining to BrdU and micronucleus assay. *Radiat Res* **125**: 243–247

Masunaga S, Ono K and Abe M (1993) The detection and modification of the hypoxic fraction in quiescent cell populations in murine solid tumors. *Br J Radiol* **66**: 918–926

Mitchell J, Morstyn G, Russo A, Kinsella T, Fornance A, McPherson S and Glatstein E (1984) Differing sensitivity to fluorescent light in Chinese hamster cells containing equally incorporated quantities of BUdR versus IUdR. *Int J Radiat Oncol Biol Phys* **10**: 1447–1451

Moulder JE and Rockwell S (1984) Hypoxic fractions of solid tumors: experimental techniques, methods of analysis, and a survey of existing data. *Int J Radiat Oncol Biol Phys* **10**: 695–712

© Cancer Research Campaign 1997

Nishimura Y, Hiraoka M, Jo S, Akuta K, Yukawa Y, Shibamoto Y, Takahashi M and Abe M (1988) Microangiographic and histologic analysis of the effects of hyperthermia on murine tumor vasculature. *Int J Radiat Oncol Biol Phys* **15**: 411–420

Nishimura Y, Ono K, Hiraoka M, Masunaga S, Jo S, Shibamoto Y, Sasai K, Abe M, Iga K and Ogawa Y (1990) Treatment of murine SCC VII tumors with localized hyperthermia and temperature-sensitive liposomes containing cisplatin. *Radiat Res* **122**: 161–167

Oleson JR, Samulski TV, Leopord KA, Clegg ST, Dewhirst MW, Dodge RK and George SL (1993) Sensitivity of hyperthermia trial outcomes to temperature and time: implications for thermal goals of treatment. *Int J Radiat Oncol Biol Phys* **25**: 289–297

Oleson JR (1995) Hyperthermia from the clinic to the laboratory: hypothesis (Eugene Robinson Special Lecture). *Int J Hyperthermia* **11**: 315–322

Ono K, Wandl EO, Tsutsui K, Sasai Y and Abe M (1989) The correlation between cell survival curve and dose response curve of micronucleus (MN) frequency. *Strahlenther Onkol* **165**: 824–827

Overgaard J (1989) Sensitization of hypoxic tumor cells – clinical experience. *Int J Radiat Biol* **56**: 801–811

Overgaard J, Gonzales DG, Hulshor MCCM, Archangeli G, Dahl O, Mella O and Bentzen SM (1995) Randomized trial of hyperthermia as adjuvant to radiotherapy for recurrent or metastatic malignant melanoma. *Lancet* **345**: 540–543

Secomb TW, Hsu R, Ong ET, Gross JF and Dewhirst MW (1995) Analysis of the effect of oxygen supply and demand on hypoxic fraction in tumors. *Acta Oncol* **34**: 313–316

Song CW, Shakil A, Osborn JL and Iwata K (1996) Tumour oxygenation is increased by hyperthermia at mild temperatures. *Int J Hyperthermia* **12**: 367–373

Steel GG (1977) Cell population kinetics of tumors in experimental animals. In *Growth Kinetics of Tumours*, Steel GG (ed.), pp. 146–216. Clarendon Press: Oxford

Valdagni R, Liu F-F and Kapp DS (1988) Important prognostic factors influencing outcome of combined radiation and hyperthermia. *Int J Radiat Oncol Biol Phys* **15**: 959–972

Vaupel P (1990) Pathological mechanisms of hyperthermia in cancer therapy. In *Biological Basis for Oncologic Thermotherapy*, Gautherie M (ed.), pp. 73–134. Springer: Heidelberg

© Cancer Research Campaign 1997